# The yearly financing need of providing paid maternity leave in the informal sector in Indonesia

**DOI:** 10.1186/s13006-021-00363-7

**Published:** 2021-02-15

**Authors:** Adiatma Y. M. Siregar, Pipit Pitriyan, Donny Hardiawan, Paul Zambrano, Mireya Vilar-Compte, Graciela Ma Teruel Belismelis, Meztli Moncada, David Tamayo, Grace Carroll, Rafael Perez-Escamilla, Roger Mathisen

**Affiliations:** 1 Center for Economics and Development Studies, Department of Economics, Faculty of Economics and Business, Universitas Padjadjaran, Jl. Hayam Wuruk 6 – 8, West Java 40115 Bandung, Indonesia; 2 Center for Health Technology Assessment (CHTA), Universitas Padjadjaran, West Java Bandung, Indonesia; 3 West Java Development Institute (INJABAR), Universitas Padjadjaran, West Java Bandung, Indonesia; 4 Alive & Thrive, FHI 360, Southeast Asia, 7F, Opera Business Center, 60 Ly Thai To Street, Hanoi, Vietnam; 5 Research Institute for Equitable Development (EQUIDE), Mexico City, Mexico; 6 Yale School of Public Health, Connecticut New Haven, USA

**Keywords:** Informal sector, Breastfeeding, Maternity protection, Maternity leave, Costing, Maternity cash transfer, Indonesia

## Abstract

**Background:**

The economic cost of not breastfeeding in Indonesia is estimated at US$1.5–9.4 billion annually, the highest in South East Asia. Half of the 33.6 million working women of reproductive age (WRA) in Indonesia (15–49 years) are informal employees, meaning they are working as casual workers or they are self-employed (small scale business) and assisted by unpaid/family worker(s). No specific maternity protection entitlements are currently available for WRA working informally in Indonesia. This study aims to estimate the financing need of providing maternity leave cash transfer (MCT) for WRA working in the informal sector in Indonesia.

**Method:**

The costing methodology used is the adapted version of the World Bank methodology by Vilar-Compte et al, following pre-set steps to estimate costs using national secondary data. We used the 2018 Indonesian National Socio-Economic Survey to estimate the number of women working informally who gave birth within the last year. The population covered, potential cash transfer’s unitary cost, the incremental coverage of the policy in terms of time and coverage, and the administrative costs were used to estimate the cost of MCT for the informal sector.

**Result:**

At 100% coverage for 13 weeks of leave, the yearly financing need of MCT ranged from US$175million (US$152/woman) to US$669million (US$583/woman). The share of the yearly financing need did not exceed 0.5% of Indonesian Gross Domestic Product (GDP).

**Conclusions:**

The yearly financing need of providing MCT for eligible WRA working in the informal sector is economically attractive as it amounts to less than 0.5% of GDP nominal of Indonesia. While such a program would be perceived as a marked increase from current public health spending at the onset, such an investment could substantially contribute to the success of breastfeeding and substantial corresponding public health savings given that more than half of working Indonesian WRA are employed in the informal sector. Such policies should be further explored while taking into consideration realistic budget constraints and implementation capacity.

**Supplementary Information:**

The online version contains supplementary material available at 10.1186/s13006-021-00363-7.

## Background

Exclusive breastfeeding (EBF) is defined as the proportion of infants 0–5 months of age who received only breastmilk [1]. Around half of all Indonesian children under 6 months were not exclusively breastfed in 2017 [2]. While this figure meets the Global Nutrition Target of 50% EBF by 2025 [3], much is required to maintain and/or increase this proportion. The economic cost of not breastfeeding in Indonesia is estimated to be as high as US$1.5–9.4 billion annually, the highest in South East Asia [4–6]. The costs of not breastfeeding estimates include costs of treating diarrhea, respiratory disease, ovarian cancer, type 2 diabetes, income loss due to lower cognitive development, and current and future mortality.

Maternity protection policies that include paid maternity leave are crucial to ensure the health of mothers and children and that women meet their breastfeeding goals [7–12]. Maternity protection allows mothers to be economically active while ensuring the safety and success of their pregnancy, and caregiving of their children, including breastfeeding [13]. Maternity leave itself is associated with higher rates of breastfeeding in low- and middle-income countries, and provides broad social, developmental, and health benefits for working mothers and newborns, as well as promoting gender equity. Such benefits include alleviating the costs of sickness, cognitive losses and deaths due to not breastfeeding [4–6, 14]. Providing paid maternity leave entitlements for working women may also be useful to improve maternal-child physical and mental health and family wellbeing, and also to potentially increase women’s participation in the labour market [8, 11, 12, 15, 16]. Studies have indeed shown that paid maternity leave may improve breastfeeding outcomes; mothers receiving paid leave for more time breastfeed longer [9, 11, 17–20]. Although the empirical evidence on the impact of maternity cash transfer (MCT) on breastfeeding outcomes is still limited, there are strong reasons to expect that maternity benefits, including MCTs, are needed to improve breastfeeding among women employed in the informal economy. Specifically, UNICEF’s cash transfer conceptual framework posits that social cash transfers can lead to higher EBF rates as the mother would be empowered to have more time for childcare [21]. Indeed, UNICEF reports qualitative evidence showing that maternity benefits can empower caregivers to spend more time raising their children [22].

About half of women in Indonesia are in the workforce [23], thus it is crucial to develop policies to ensure that employed mothers are able to provide essential nurturing care both at home and while the caregiver works in the first 6 months of a child’s life without sacrificing both income and employment opportunities. Some 48% of approximately 70 million women of reproductive age (WRA) in Indonesia are in the labor force. Among this population, 52% are informal employees [24]. According to the National Labor Survey (SAKERNAS) Interviewer Guide, women can be classified as working in the informal sector if they are working as casual workers or if they are self-employed (small-scale business) and assisted by unpaid/family worker(s) [25].

Currently, maternity protection entitlements are not available for WRA working informally in Indonesia and only available for WRA working in the formal sector, calling for a reform in the current policies supporting breastfeeding [26]. However, efforts to scale-up breastfeeding support for women working in the informal sector in Indonesia have been, to some extent, covered by the existing social protection program called the Family Hope Program (*Program Keluarga Harapan*/PKH) scheme [27, 28]. This conditional cash transfer program provides a flat-rate cash transfer for the 20% poorest families with students, pregnant women or disabled family members. For a pregnant woman to participate in the program she must attend four antenatal checkups and consume iron tablets during her pregnancy, be assisted by a trained professional birth attendant during delivery, and have two post-natal care visits [29]. These measures may indirectly contribute to improving breastfeeding.

Previous studies in Indonesia have shown that the annual cost of not breastfeeding is large, ranging from 0.14 to 0.90% of Indonesia’s GDP in 2018 [4–6] and outweighs the financing need of paid maternity leave within the formal sector [30]. As women in low and middle income countries (including Indonesia) are more likely to work in the informal sector [31] and mostly are uncovered by maternity leave policies [32], paid maternity leave policies within the informal sector would potentially result in larger benefits both economically and non-economically than within the formal sector. Unfortunately, providing paid maternity leave to informal workers is still a challenge globally [32]. In Indonesia, the International Labour Organization (ILO) coverage classification put the country in the 10 to 32% range [7], showing that the coverage even in the formal sector is not yet optimal. One of the disincentives of providing paid maternity leave is perceived or actual financial cost by employers [30, 33]. Another issue is that the cost of supporting a maternity benefit for WRA working informally likely needs to be covered entirely by the government. Therefore, it is imperative to estimate the annual cost of providing maternity protection entitlements within the informal sector for advocacy purposes to create the will among decision makers to develop policies and programs to provide maternity benefits to women employed in the informal sector [34, 35]. Investing in maternity protection for mothers working informally is a form of social justice that creates better conditions for women to exercise their choice and may protect their right to breastfeed [36, 37].

This study aims to estimate the financing need of providing a maternity leave cash transfer (MCT) for WRA who work informally in Indonesia. Such studies are lacking in Indonesia and in other low- and middle-income countries all over the globe [31, 38]. Furthermore, the few studies available have analyzed cash transfers targeting several outcomes (e.g. education and health) and not specifically paid maternity leave, in spite that some of these studies have shown that cash transfers may have a positive impact on breastfeeding outcomes [39–44]. This study is the first to provide such estimates for Indonesia, and as such can provide urgently needed evidence for policy making purposes in the context of supporting recommended breastfeeding practices, especially given the relatively low health budget in Indonesia (under 5% share of GDP as of 2014) [45]. This study follows on from our previous research on the financing need to expand maternity protection for the formal sector [30] and begins to fill the gap in such estimates for informal sector maternity benefits.

## Methods

The costing methodology used is the adapted version of the World Bank methodology by Vilar-Compte et al [31], following pre-set steps to estimate costs using nationally secondary data. The following formula was used in the study:

$$ {MCT}_y=\left(\left(\alpha \ast {Pop}_y\right)\ast {UC}_{CT}\ast {IC}_y\right)+{AdmC}_y $$

Where:

MCT_y_ : the MCT annual cost for a given year of intervention

α : probability of WRA giving birth in year *y*

α x Pop_y_ : population of women of reproductive ages (i.e. 18–49 years of age) in year *y* weighted

by α

UC_CT_ : unit cost of the CT

IC_y_ : incremental coverage (IC) of MCT assumed for a year *y*

AdmC_y_ : administration cost in year *y*

We used the 2018 Indonesian National Socio-Economic Survey (SUSENAS) [24], an annual nationally representative survey able to provide population level estimates using provided weights. SUSENAS is the largest socioeconomic survey, typically comprising nationally representative samples of 200,000 households. SUSENAS includes general information and personal characteristics of respondents, as well as the variables used to determine fertility and the type of labor (i.e. formal vs. informal). In line with our study, SUSENAS enables us to estimate the number of women working informally who gave birth within the last year.

To calculate the costs in this study, the previous formula was applied through the following steps:

- *Step 1:* We computed the number of women who work informally and gave birth in the prior year, given a vector of individual characteristics (we provided more detailed explanation of the definition of informal sector as well as rural/urban in Additional file 1). Instead of an overall population estimate, it is recommended to separate the population into subgroups with different fecundity and participation in the informal sector to obtain a more accurate estimate of the target population for a given year. We separated the number of WRA working in the informal sector into several subgroups, namely age (15–19, 20–24, 25–29, 30–34, 35–39, 40–44, 45–49), education (no education, primary education, junior high school, senior high school, diploma, and university), marital status (single, married, divorced, widow), locality (urban, rural), and gave birth in the last 1 year, resulting in 308 subgroups (e.g. an example of a subgroup: the number of women working informally, aged 15–19, no education, single, living in an urban area, gave birth in the last 1 year). SUSENAS provides data on giving birth within the last 2 years, thus we divided the number by two for each of the subgroups to reflect the number of WRA who gave birth within the last 1 year.
- *Step 2:* We then calculated the percentage of WRA working informally who gave birth in the prior year per subgroup as a share of the total WRA working informally (i.e. the number of WRA working informally who gave birth in the last 1 year in a subgroup/the total number of WRA working informally) to estimate α. For each subgroup, α was defined as the probability of WRA working informally who gave birth in the last year within each of the subgroups, resulting in 308 different values for α.
- *Step 3:* We determined a realistic estimate of beneficiaries who may claim maternity leave in the informal sector in a given year by weighting the population of WRA employed in the informal sector by α (i.e. probability of having a child in a given year). *Pop*_*y*_ or WRA data at the population level were obtained from World Bank estimate [46], adjusted by the percentage of female labor participation rate and adjusted further by WRA who work informally using SUSENAS data [24]. *Pop*_*y*_ was then multiplied by α of the respective subgroups to determine the number of WRA who work informally and gave birth within the prior year (*α* ∗ *Pop*_*y*_).
- *Step 4:* The unit cost data (UC) was proxied by, first, minimum wage data (average minimum wage derived from various documents at the provincial level depicting respective minimum wage); second, unit cost of a cash transfer program called Family Hope Program (PKH) [47, 48]; and third, the poverty line (derived from World Bank report) [49]. UC was multiplied by results from step 3: (*α* ∗ *Pop*_*y*_) ∗ *UC*_*CT*_.
- *Step 5:* Incremental coverage (IC) was determined based on regulations, recommendations, and literature regarding the length of leave and coverage. The length of leave used in this study started from the application of the current Indonesian law of 13 weeks maternity leave (approximately 3 months leave) [26], and increased to 14 (minimum requirement of ILO) [50], 18 (extension according to ILO) [51], and 26 weeks (WHO recommendation) [3]. We also used two coverage scenarios of WRA working informally eligible for maternity leave, namely 21% (a midrange value from the ILO coverage classification placing Indonesia in the 10 to 32% level) [7] and 100%. These were then multiplied by step 4: (*α* ∗ *Pop*_*y*_) ∗ *UC*_*CT*_ ∗ *IC*.
- *Step 6:* As this type of cash transfer (CT) would be new, the administrative cost needs to be added. Administrative cost (*AdmC*_*y*_) was derived based on a previous study of the national Family Hope Program (PKH), managed by The Ministry of Social Affairs. The program provides the lowest 20% income household group with conditional cash transfers (CCT) to increase its family members’ access to health and education facilities. We believe this program approximates the context simulated in our MCT study for WRA working informally. The simulation approach was needed since no actual MCT programs for women working informally exist. The administration cost of PKH is deemed moderate and the program has a better administrative and management structure compared to other CCT programs in Indonesia. The share of PKH administrative cost (14% in 2009) is closer to other mature CCT programs in other countries (around 8%) [29, 52–56]. In monetary terms, the average administrative costs per household beneficiaries in 2010 was about US$24 [29]. We converted this value into 2018 value using Consumer Price Index obtained from World Bank data [57] resulting in a fixed cost of US$35 per person. To calculate the total administrative cost, the fixed cost per person was multiplied by (*α* ∗ *Pop*_*y*_): *US$*35 ∗ (*α* ∗ *Pop*_*y*_) = *AdmC*_*y*_. Using this cost, the percent of our administrative cost as compared to the total cost falls between 5 and 36% (Table 3), depending on the UC used in the calculation. Our administrative cost per woman and its share out of the total cost is higher than that of Mexico, but comparable to the study conducted in the Philippines [31, 58].

The administrative cost (*AdmC*_*y*_) was added to the total cost obtained from step 5 to yield the total cost of providing cash transfers to WRA working informally. The cost per women was calculated by dividing the total cost by the estimated number of women expected to receive maternity leave. The details of the assumptions used for our calculations are provided in Table 1. All costs were converted to USD using the 2019 reference exchange rate from Bank of Indonesia [59].

**Table 1:** Assumptions and values used in the analysis

Items	Value used in base scenario	Sources
Exchange rate (2019)	Rp 14,236/US$	Bank of Indonesia [59]
Rate of cash benefit provided to employees by employers (%)	100	ILO [7]
Minimum wage per month (US$)^a^	159.20 (39.80/week)	
2/3 of minimum wage per month (US$)^a^	106.13 (26.53/week)	
Family Hope cash transfer per month [47, 48]	168.59 (42.15/week)	
Poverty line per month (3.2US$ PPP 2011 per day, converted into 2018 nominal value using PPP conversion of Rp5,341.5/US$ and 2019 exchange rate)	36.02 (9.01/week)	The World Bank [49], Ministry of National Development Planning of Republic of Indonesia [60]
Number of WRA (15–49 years)	71,182,875	The World Bank [46]
Percentage of working WRA (%)	50.17	National Bureau of Statistics Indonesia [24]
Percentage of women working in the informal sector (out of working WRA) (%)	59.11	National Bureau of Statistics Indonesia [24]
Potential coverage of women working in the informal sector potentially eligible to receive paid maternity leave (%)	21^c^ and 100	ILO [7]
Length of maternity leave (weeks)	13, 14, 18 and 26	Ministry of Manpower and Transmigration of Republic of Indonesia [26], WHO [3]
Administration cost per female covered (US$)^b^	35 (2018)	The World Bank [29]
Indonesian GDP nominal 2018 (US$)	1,042,173,300,000	The World Bank [61]

^a^The wage reflects average provincial minimum wage, compiled from various provincial regulation documents; ^b^assumed to be similar to the Family Hope Program [29], adjusted to 2018 value using CPI of 147% (2010 = 100) [57]; ^c^Mean of coverage in law of maternity leave [7]

This table shows all of the assumptions and values used in the calculation

## Results

Table 2 presents the characteristics of WRA in Indonesia who work informally and gave birth, using SUSENAS data. As many as 71.1 million females were categorized as WRA, and of this amount 50.17% were working, and among those, 59.11% were working informally. Of WRA working informally, 5.43% gave birth within the last 1 year. Based on the calculation of coverage (21 and 100%, Table 1) multiplied by the number of informally working women, there are 240,913 (21% coverage) and 1,147,204 (100% coverage) women who would be potentially eligible to receive the MCT program.

**Table 2:** Characteristics of informally working WRA in Indonesia

Variables	Categories	Work informally (%)^a^	Gave birth within the last year (%)^a^
Age group (years)	15–19	53.0	3.8
	20–24	37.9	9.9
	25–29	47.4	12.0
	30–34	57.4	9.1
	35–39	63.8	5.9
	40–44	68.0	2.3
	45–49	70.2	0.6
Education level	No education, kindergarten or incomplete elementary school	83.1	5.1
	Elementary school	79.1	4.4
	Junior high school	70.6	5.6
	Senior high school	51.1	6.4
	Vocational school	19.6	8.0
	University	12.5	8.7
Marital status	Single	32.8	0.0
	Married	64.8	6.4
	Divorced	52.0	2.8
	Widowed	68.4	1.5
Type of locality	Urban	41.8	5.2
	Rural	72.4	5.6

Source: SUSENAS 2018 [24], ^a^out of working WRA

This table shows the characteristics of WRA working informally using the SUSENAS data

### The annual financing need for MCT in the informal sector

Table 3 provides the cost calculation based on the formula presented in the methods section using the different unit costs, at 21 and 100% coverage. The table showing the costs per province is presented in Appendix A. Understandably, the highest total costs are associated with the total cost based on the minimum wage and the unit cost of MCT per month, the greatest unit cost. The administrative cost (similar for all three UCs) was added to each of the four different UCs to estimate the total cost of MCT for eligible informally working WRA. At 100% coverage, the total cost calculated by using minimum wage, 2/3 minimum wage, PKH cash transfer, and poverty line as the UC for 13 weeks amounted to around US$634million (US$553/woman), US$436million (US$380/woman), US$669million (US$583/woman), and US$175million (US$152/woman), respectively. The comparison between UC for respective coverage (100% or 21%) is only differentiated by the UC as the other variables are constant, including the administration cost. The costs at 21% coverage for any length of maternity leave are 5 times lower than the estimates at 100% coverage. Although the cost per woman could be about 11 times higher than the health expenditure per capita in Indonesia in 2014 (adjusted to 2018 value), the estimate did not exceed 0.5% of 2018 nominal GDP [45, 57, 61].

**Table 3:** The yearly financing need of MCT in the informal sector

Type of UC/% and length of coverage (weeks)	Number of WRA working informally covered	Cost of MCT (US$)	Administrative cost (US$)	Total cost (US$)	% of GDP 2018 (nominal)	Cost per woman (US$)
*100% coverage*						
Minimum wage						
13	1,147,204	593,551,960	40,390,767	633,942,726	0.061	553
14	1,147,204	639,209,803	40,390,767	679,600,569	0.065	592
18	1,147,204	821,841,175	40,390,767	862,231,942	0.083	752
26	1,147,204	1,187,103,919	40,390,767	1,227,494,686	0.118	1070
2/3 minimum wage						
13	1,147,204	395,701,306	40,390,767	436,092,073	0.042	380
14	1,147,204	426,139,868	40,390,767	466,092,073	0.045	407
18	1,147,204	547,894,116	40,390,767	588,284,883	0.056	513
26	1,147,204	791,402,613	40,390,767	831,793,380	0.080	725
PKH Cash transfer						
13	1,147,204	628,560,907	40,390,767	668,951,674	0.064	583
14	1,147,204	676,911,746	40,390,767	717,302,513	0.069	625
18	1,147,204	870,315,102	40,390,767	910,705,869	0.087	794
26	1,147,204	1,257,121,814	40,390,767	1,297,512,581	0.125	1131
Poverty line						
13	1,147,204	134,298,323	40,390,767	174,689,090	0.017	152
14	1,147,204	144,628,964	40,390,767	185,019,731	0.018	161
18	1,147,204	185,951,525	40,390,767	226,342,292	0.022	197
26	1,147,204	268,596,647	40,390,767	308,987,414	0.030	269
*21% coverage*						
Minimum wage						
13	240,913	124,645,912	8,482,061	133,127,973	0.013	553
14	240,913	134,234,059	8,482,061	142,716,120	0.014	592
18	240,913	172,586,647	8,482,061	181,068,708	0.017	752
26	240,913	249,291,823	8,482,061	257,773,884	0.025	1070
2/3 minimum wage						
13	240,913	83,097,274	8,482,061	91,579,335	0.009	380
14	240,913	89,489,372	8,482,061	97,971,433	0.009	407
18	240,913	115,057,764	8,482,061	123,539,826	0.012	513
26	240,913	166,194,549	8,482,061	174,676,610	0.017	725
PKH Cash transfer						
13	240,913	131,997,790	8,482,061	140,479,852	0.013	583
14	240,913	142,151,467	8,482,061	150,633,528	0.014	625
18	240,913	182,766,171	8,482,061	191,248,233	0.018	794
26	240,913	263,995,581	8,482,061	272,477,642	0.026	1131
Poverty line						
13	240,913	28,202,648	8,482,061	36,684,709	0.004	152
14	240,913	30,372,082	8,482,061	38,854,143	0.004	161
18	240,913	39,049,820	8,482,061	47,531,881	0.005	197
26	240,913	56,405,296	8,482,061	64,887,357	0.006	269

This table shows the costs calculation of financing MCT in informal sector per year

## Discussion

This study estimates the annual financing need of providing an MCT in the informal sector. The annual financing need of providing an MCT for all WRA working informally ranges from US$175 million (US$152/woman) to US$1.3 billion (US$1131/woman) depending on the UC applied. At 100% coverage, the total financing need of providing MCT for WRA working informally is much higher than the existing CT program (PKH). As previously described, the PKH program provides the lowest 20% income household group with conditional cash transfers (CCT) to increase its family members’ access to health and education facilities, to improve maternal and child health, and it is the closest type of existing CT program in Indonesia to our proposed MCT program. The annual cost of PKH adjusted to 2018 value is US$209million, covering 778,000 households in 2010 [29, 57]. At 100% coverage, our MCT program total cost using CT as UC (for 13 weeks leave) amounts to around US$669million and US$1.3 billion (26 weeks leave). Using other UCs, except for the poverty line at 13 and 14 weeks, all total costs at 100% coverage are higher than PKH. At the lower coverage rate of 21% the cost is much lower (US$140million for 13 weeks leave, using CT as UC), similar to the other total costs estimated by using other UCs at 21% coverage. As such, a trade off occurs between increasing coverage or producing a more feasible total expenditure.

The PKH is an established program producing positive results (e.g. increased utilization of childbirth through trained health professionals, stunting reduction) [28]. The introduction of MCT in the informal sector may require significant advocacy to convince policy makers of the importance of the transfer program to implement at 100% coverage for 26 weeks. Given budget constraints can be one of the obstacles for implementing maternity protection policies [30, 45], the initial introduction of MCT for the informal sector could start at a lower cash transfer benefit level and/or coverage (i.e. 13 weeks and/or 21% coverage), using a more moderate UC (i.e. poverty line or 2/3 minimum wage), and increase time/benefit provided, coverage, and UC gradually as implementation progresses. However, further studies are also required to determine the minimum cash transfer amount needed to improve health outcomes and related behaviors such as breastfeeding. As PKH has already yielded positive results, the PKH cash transfer unit cost can be considered as a tentative benchmark of the required minimum cash transfer amount.

We also found that our total financing need estimates in all scenarios did not exceed 0.5% of Indonesia nominal GDP in 2018, a much lower percentage than the share of health expenditure on GDP. The cost per woman, however, could be around 11 times higher than the health expenditure per capita [45] and 8 times higher than the cost of PKH per household [29]. Thus, although the financing need seems low in comparison to the total GDP, the cost per woman may not look appealing to policy makers. This can be challenging since budget availability has already been recognized as one of the issues faced in optimizing the more established paid maternity leave policy for the formal sector [30]. As MCT policies for informal workers currently do not exist, this challenge will require proper program and financial planning as well as support from the government and relevant stakeholders since even now the local government struggles with allocating its budget to support the policy for the formal sector, let alone the informal sector. Additionally, even though the policies regulating maternity leave are available for the formal sector, its implementation is still not optimal [62–64]. This may prove to be a challenge for the informal sector to develop and implement MCT policy. If such policies are to be implemented, it should ensure that women are able to access MCT without facing the risk of discrimination due to the policy implementation [65, 66].

One aspect that should be advocated to policy makers if MCT policies are to be optimally implemented for both formal and informal sectors is that the cost of not breastfeeding is much higher than the financing need of implementing MCT policy. The cost of not breastfeeding in Indonesia includes the irreversible costs due to sickness and cognitive loss which may be higher than US$1.5–9.4 billion annually, as well as the high annual level of maternal and infant deaths which may reach more than 7000 deaths [4–6]. These negative impacts of not breastfeeding should be a primary consideration in developing sound MCT policies for both the formal and informal sectors. Indeed paid parental leave has been shown to support meeting the Sustainable Development Goals (SDGs) outcomes such as lower infant mortality, increased exclusive breastfeeding rate, and better economic outcomes for women [12]. The total financing need of both our estimate for the informal sector, and the other estimate from the previous study on the formal sector [30] shows that the combined financing need of providing MCT to eligible WRA in both the formal and informal sectors at 100% coverage based on minimum wage amount to be around US$2 billion per year, roughly 4.5 times lower than the estimate of the cost of not breastfeeding in Indonesia. This indicates the value of investing in MCT, in addition to its benefits in terms of alleviating the costs of sickness, cognitive losses and deaths due to not breastfeeding and improving maternal-child physical and mental health and family wellbeing, and also to potentially increasing women’s participation in the labour market [4–6, 8, 11, 12, 14–16, 67]. However, the proposed MCT approach would require, among other things, sound monitoring to ensure that breastfeeding actually took place, consistency in the best timing of delivery of cash distribution and breastfeeding counselling visits, recognizing that many mothers receiving the cash transfer face major social determinants of health challenges. These need to be addressed through supportive social protection, efficacy evaluation of the intervention, and economic policies [41, 42, 68–70]. In addition, it should also be stressed that there is evidence showing that maternity leave schemes have other benefits in addition to breastfeeding such as a larger share of women returning to work [71], improved mothers’ mental health [67], and lower neonatal mortality [72]. While these additional benefits have been reported in formal maternity leave schemes, they will need to be considered when evaluating the efficacy and social return of MCT.

As most working WRA in Indonesia are working in the informal sector, providing MCT to this group may reduce the cost of not breastfeeding in Indonesia by a large number. Other barriers to providing effective maternity protection policies such as strong breastmilk substitutes marketing, government budget constraints, perceived or actual financial cost by employers (thus reducing their profits), absenteeism, lack of information on and support for maternity protection, lack of workplace lactation rooms, and socio-cultural factors (e.g. the need to introduce complementary food early) [9, 30, 33] should also be addressed adequately to ensure the success of any maternity protection policies [35].

This study has a few limitations that need to be addressed through further research to reduce the uncertainties around our costing estimates. First, using PKH cash transfer UC is not a perfect comparison for assessing the idea of providing maternity leave CT to informally working WRA. PKH is targeted at families in the 20% lowest income bracket with, among others, pregnant women as a family member. However, this was our only modeling option as currently this is the only cash transfer program that targets families with pregnant women to promote maternal health for the poor. In addition, we used alternative operationalizations of UC to anticipate for cost differences. Second, our study draws on national level data which may not accurately represent unique local characteristics. This is quite important since regions across the Indonesian archipelago have diverse characteristics which may result in different estimates of costs for maternity protection policies (e.g. higher MCT due to the need to pay for a more expensive transport mode to reach a health facility). Thus, future studies may explore sub-national costs and breastfeeding practices and develop a more locally representative result as a basis for a local maternity protection policy. Also, since our study only focuses on Indonesia, a comparative study with other countries with roughly similar settings would be useful for comparison to develop a more comprehensive cost analysis. Third, the administrative costs are a rough estimate that may have biases. As more countries implement such maternity leave CT, better estimations should be available in the future. Last, although studies have shown the positive impacts of paid maternity leave in the formal sector, including improved breastfeeding outcomes; more research is needed to confirm the effectiveness of MCT on improving breastfeeding. Prospective studies are urgently needed in this area.

## Conclusion

The yearly financing need of providing MCT for eligible WRA working in the informal sector is significantly lower than the current annual cost of not breastfeeding in Indonesia, as computed in previous work [4–6]. While this program would represent a marked increase in current public health spending at the onset, the total financing need estimates in all scenarios are less than 0.5% of the country’s 2018 nominal GDP. More than half of working Indonesian WRA are employed in the informal sector, thus an MCT program targeting this sector could have a substantial impact on breastfeeding rates in the country. These policies have the potential to contribute to the success of breastfeeding and as a result help avoid some infant and mother deaths and improve health, social, and economic sectors. However, challenges such as budget constraints and less than optimal policy implementation must be addressed to devise an effective and realistic strategy for MCT implementation and enforcement based on sound implementation science methods [73].

## Supplementary Information

**Additional file 1.** Definition of informal sector and rural/urban. This description shows the definition of informal sector as well as the definition of rural/urban used in this study.

## Data Availability

The SUSENAS data are available from Statistics Indonesia (BPS) repositories by request. All calculation data generated or analyzed during the current study are available from the corresponding author on reasonable request.

## Abbreviations

CCT: conditional cash transfers
EBF: Exclusive breastfeeding
GDP: Gross Domestic Product
IC: incremental Coverage
ILO: International Labour Organization
MCT: maternity leave cash transfer
PKH: *Program Keluarga Harapan*/Family Hope Program
SDGs: Sustainable Development Goals
SUSENAS: *Survey Sosial Ekonomi Nasional*/National Socio-Economic Survey
UC: unit cost
WRA: women of reproductive age
