# Supplementary material for: The yearly financing need of providing paid maternity leave in the informal sector in Indonesia
Source: Int Breastfeed J. 2021 Feb 15;16:17. doi: 10.1186/s13006-021-00363-7 (PMC7885595; doi:10.1186/s13006-021-00363-7)
Supplement: Supplementary file 1 — Additional file 1. Definition of informal sector and rural/urban. This description shows the definition of informal sector as well as the definition of rural/urban used in this study. [file 13006_2021_363_MOESM1_ESM.docx]

**Additional file 1. Definition of informal sector and rural/urban**

**Definition of informal sector**

Although we use SUSENAS as the main source of the data, the term of informal workers refers to the National Labor Survey (SAKERNAS) definition since the variable definition and coding are similar in both statistics. Based on the SAKERNAS’s Interviewer Guide, there are three approaches in classifying informal workers. Firstly, it is based on individual’s working status. Informal workers are those who are working as casual workers or self-employed assisted with unpaid worker(s). Secondly, by combining the information of working status and occupational status. For example, productive workers with the status of self-employed assisted by unpaid workers are categorized as formal workers. Thirdly, it is based on International Conference of Labor Statisticians (ICLS) classification, in which informal workers are those are working outside the government/institution/profit or non-profit organization/cooperative, or individuals or household business sector equipped with simple financial accounting or none financial accounting. We decided to employ the first term with the purpose of statistical consistency. Up to now, the majority of the Government of Indonesia’s publications related to informal/formal workers are based on working status.

**Definition of rural/urban**

Rural and urban could be defined based of its function, administrative factors, or expert judgment. For this study, we followed the Statistics Indonesia Office (BPS) classification of urban and rural, which is based on the Decree of the Head of BPS No. 37 year 2010 on the Classification of Rural and Urban in Indonesia. According to the Decree, rural is an administrative status of an area at sub-district level which does not meet the criterion of urban. Among criterion to be fulfilled by an area to be defined as urban are: population density, share of farm households, and the availability of access to urban facilities. Applying score for each of the factor, urban is an area with the weighted score above 10, while rural is an area scored less than 10. According to these scores, there are 15,786 urban and 61,340 rural in Indonesia in 2010.
